# Occludin: a gatekeeper of brain Infection by HIV-1

**DOI:** 10.1186/s12987-023-00476-7

**Published:** 2023-10-16

**Authors:** Silvia Torices, Leah Daire, Sierra Simon, Oandy Naranjo, Luisa Mendoza, Timea Teglas, Nikolai Fattakhov, Daniel Adesse, Michal Toborek

**Affiliations:** 1 Department of Biochemistry and Molecular Biology, University of Miami School of Medicine, 528E Gautier Bldg. 1011 NW 15th Street Miami, Miami, FL 11336 USA; 2 Laboratório de Biologia Estrutural, Instituto Oswaldo Cruz, Fiocruz, Rio de Janeiro, Brazil

**Keywords:** Occludin, HIV, Blood brain barrier, Virus

## Abstract

Compromised structure and function of the blood-brain barrier (BBB) is one of the pathological hallmarks of brain infection by HIV-1. BBB damage during HIV-1 infection has been associated with modified expression of tight junction (TJ) proteins, including occludin. Recent evidence indicated occludin as a redox-sensitive, multifunctional protein that can act as both an NADH oxidase and influence cellular metabolism through AMPK kinase. One of the newly identified functions of occludin is its involvement in regulating HIV-1 infection. Studies suggest that occludin expression levels and the rate of HIV-1 infection share a reverse, bidirectional relationship; however, the mechanisms of this relationship are unclear. In this review, we describe the pathways involved in the regulation of HIV-1 infection by occludin. We propose that occludin may serve as a potential therapeutic target to control HIV-1 infection and to improve the lives of people living with HIV-1.

## Introduction

Human immunodeficiency virus (HIV-1) infection leads to a weakened immune system, causing people living with HIV-1 (PLH) to become susceptible to other pathogens. When the immune system becomes ineffective, an individual may develop acquired immunodeficiency syndrome (AIDS). In 2022, ~ 39 million people were living with HIV-1 and approximately 630,000 individuals had AIDS-related deaths. Since the start of the HIV-1 epidemic, tens of millions of people have died from HIV-1 and AIDS-related complications [1]. Due to the development of antiretroviral therapy (ART) in 1996, HIV-1 infection is now classified as a chronic, rather than fatal, disease, and the majority of affected individuals have manageable infections without AIDS development [1–3].

**Fig. 1:**
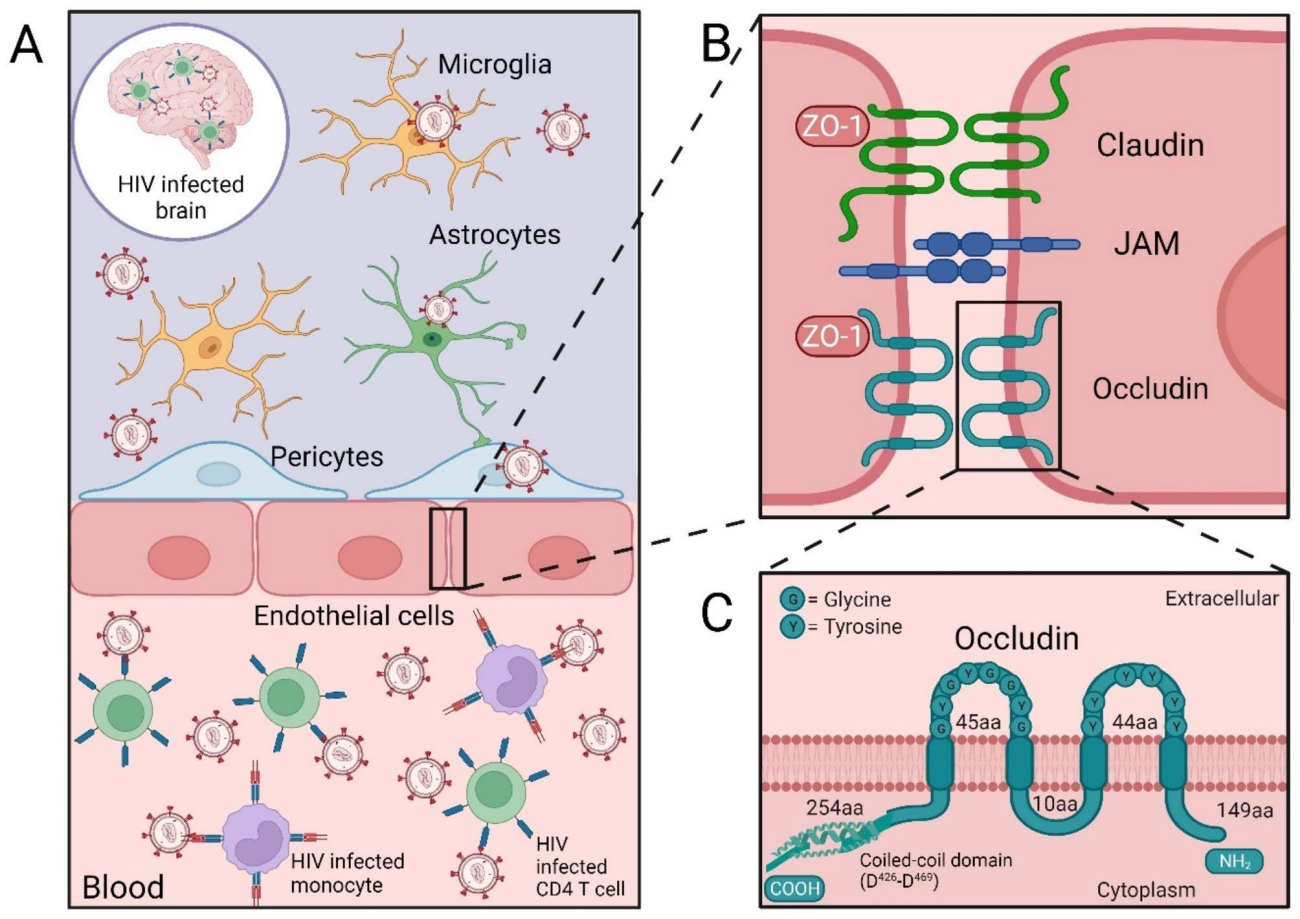
**Blood Brain Barrier (BBB) in HIV-1 infected brain and occludin structure. (A)** Schematic representation of CNS invasion by HIV-1. After infecting leukocytes in the blood (monocytes and T-cells), HIV-1 can cross the BBB via the Trojan horse mechanism and infect various CNS cells, such as astrocytes, pericytes, and microglia cells. Moreover, brain infection by HIV-1 is associated with a disruption of the BBB integrity by altering tight junction (TJ) protein expression and function. **(B)** Schematic representation of the TJs formed by transmembrane proteins (e.g., occludin, claudins, and junctional adhesion molecules [JAM]). **(C)** Schematic representation of occludin structure, showing the domains and phosphorylation residues

Early after infection, HIV-1 enters the central nervous system (CNS) [4] (Fig. 1A). While the mechanisms of this process are still not fully understood, the most likely is a “Trojan horse” mechanism via HIV-1 infected monocytes or T-cells, which act as carriers, allowing the virus to pass the blood-brain barrier (BBB) and infect cells of the CNS. Among the CNS cells, microglial cells, perivascular macrophages, astrocytes, and pericytes have been identified as possible reservoirs for HIV-1 [5–10]. Although the use of ART suppresses the replication of the HIV-1 virus, the main limitations of this therapy arise due to limited ability to effectively bypass the BBB. In addition, antiretroviral drugs are being transported out of the brain parenchyma by transporter systems. This inability of ART to accumulate in the CNS contributes to HIV-1 infection in the brain, the formation of viral reservoirs, and the development of cerebrovascular comorbidities [11, 12].

Formed by endothelial cells (EC), astrocytes, neurons, pericytes and microglia cells, the BBB forms a highly specialized barrier that selectively divides the brain parenchyma from the systemic blood circulation [13]. One of the main structural features of the BBB is the presence of tight junctions (TJs) formed by transmembrane proteins, such as occludin, junctional adhesion molecules (JAMs), and claudins (Fig. 1B) that interact with TJ-associated proteins, such as the scaffolding proteins *zonula occludens* (ZO) 1 or 2. During HIV-1 infection, structural modifications of the BBB have been associated with changes in the expression of TJ proteins. Recently, occludin has attained additional importance, not only for its role in maintaining the integrity of TJs, but also for its influence on cellular metabolism and regulation of HIV-1 infection [10, 14–16].

### Occludin structure

Occludin was the first transmembrane TJ protein identified [17]. Occludin is a member of the TJ-associated-MARVEL (**M**yelin/lymphocyte **A**nd **R**elated proteins for **VE**sicle trafficking and membrane **L**ink) proteins (TAMPs) family of TJ proteins containing a MARVEL-motif which consists of four transmembrane helices [18, 19]. Occludin is known as MARVEL D1, and additional proteins in this family are MARVEL D2 (tricellulin) and MARVEL D3 [20, 21]. Studies propose that the MARVEL motif may be responsible for occludin dimerization and localization to the basolateral membrane [18]. Although the MARVEL proteins have not been found to be essential to TJ formation, they appear to be important for maintaining the permeability properties of the BBB [22]. Although usually classified as being important to TJ assembly, function, and regulation, its various roles in cellular activities are unclear. For example, occludin-deficient mice maintain normal paracellular permeability and normal TJs [23]. Occludin is a 65-kDa integral plasma membrane protein containing 522 amino acids (aa) [17, 24]; however, there is evidence of additional isoforms formed by alternative splicing [25]. Occludin exhibits distinct domains: (a) a long C-terminal cytoplasmic domain (257 aa); (b) four transmembrane domains, including TM1 (23 aa), TM2 (25 aa), TM3 (25 aa), and TM4 (22 aa); (c) two extracellular loops, EL1 (46 aa), which is enhanced with tyrosine and glycine residues, and EL2 (48 aa), which includes two cysteines; (d) one intracellular loop (10 aa); and (e) a short N-terminal cytoplasmic domain (66 aa) [16, 21] (Fig. 1C). The long C-terminal domain accounting for almost 50% of its weight and which ends in the coiled coil, structurally distinguishes occludin from claudins. The N- and C-terminal regions are locations for key occludin phosphorylation residues and provide the functional variability of occludin at TJs [26]. The N-terminal domain bears a Type I WW binding motif (PPXY) and interacts with ITCH, an E3 ubiquitin protein ligase [27]. Modifications near the N-terminus affect TJ localization [28] and barrier properties [29]. The C-terminal domain contains an α-helical coiled-coil structure of approximately 426–469 aa, interacting as a dimerized four-helical bundle. This structure allows one occludin molecule to interact with another and establishes specific interactions with other regulatory proteins. Reportedly, C-terminal interactions with regulatory molecules play an important role in TJ assembly and function [30]. Despite some in vitro evidence indicating occludin N-terminal phosphorylation [31], more evidence supports the existence of C-terminal phosphorylation [26].

### Cells and organs expressing occludin

Occludin is ubiquitously expressed; however, it is present in various amounts in different tissues and cells. For example, a mouse study showed that occludin mRNA is expressed at similar levels in the duodenum, ileum, liver, and lung, with lower amounts in the brain, and higher amounts in the colon. Protein and mRNA expression levels were found to be mostly consistent across tissues, except that kidney cells produced significantly lower levels of occludin than other organs, including the brain [32]. In the human brain, elevated occludin expression in astrocytes, oligodendrocytes, and cerebral cortex pyramidal neurons was detected in both Alzheimer’s disease and vascular dementia [33]. Occludin levels were found to be inducible by TNF-α treatments in a variety of cell types [34, 35], suggesting the existence of common molecular pathways for occludin upregulation upon inflammatory stimuli. Additionally, a high degree of homology in occludin was reported across animal species [21]. Examples of other cell types expressing occludin include mouse hepatocytes [36] and activated T-lymphocytes [37]. A low-level mRNA occludin expression was also detected in HEK293 cells [38].

Regarding the BBB, occludin is expressed primarily in brain endothelial cells. Moreover, occludin expression was found to be enhanced in mouse brain endothelial cells when cocultured with resting microglia [39, 40]. Similarly, occludin levels were significantly increased in cocultures of rat endothelial cells with astrocytes [41]. Occludin is also expressed in mouse astrocytes and neurons, in addition to epithelial and endothelial tissue [42]. Several studies have shown that human brain pericytes express occludin [6, 9, 14, 43]. In addition, rat pericytes induce the expression of occludin through the release of angiopoietin via the pericyte-derived multimeric angiopoietin-1/Tie-2 pathway [39]. Taken together, occludin expression shares significant relationships among cells from various tissues, including the cells composing the BBB.

### Occludin and HIV-1 Infection

Impairment in TJ expression levels and damage to BBB permeability are associated with infection by a variety of viruses, such as Zika virus [44], human T-cell leukemia virus (HTLV-1) [45], mouse adenovirus type 1 (MAV-1) [46], and HIV. Besides its role in maintaining BBB integrity, occludin has also been characterized to play important roles in the entry and progression of several viral infections. In influenza/H1N1 [47] and HIV [14] infections, a decrease in occludin levels has been shown to increase infection. On the other hand, the opposite effect has been observed in Hepatitis C infection, where occludin was demonstrated to be an essential factor for viral entry and allowing cells to be infected [48–50]. In this review we will focus on the role of occludin in HIV infection.

Recent evidence indicates a bidirectional connection between HIV-1 infection and changes in occludin protein expression levels, pointing to occludin as a critical regulator in HIV-1 infection [14]. In this regard, the effect of the HIV-1 transactivator protein (Tat), which recruits elongation factors for RNA polymerase II, has been shown to decrease occludin expression levels in human endothelial cells [51–53]. Additional studies have used transgenic rats to demonstrate that HIV-1 proteins decrease occludin levels in the hippocampus and in epithelial cells [54, 55]. In human brain pericytes, a dual-stage response pattern has been identified, characterized by a significantly decreased occludin expression in pericytes 48 h post-infection, i.e., at the peak of active infection in these cells, followed by subsequent increased occludin levels during the development of latent infection [7, 9, 14]. The involvement of occludin in the regulation of HIV-1 replication has been confirmed in human monocytic U-937 cells, human macrophages, and HEK 293 cells [14]. To illustrate this effect, occludin silencing resulted in a 75% and 250% increase in HIV-1 replication in human primary macrophages and differentiated monocytic U937 cells, respectively [56]. In contrast, occludin overexpression in HIV-infected human brain pericytes decreased the rate of HIV-1 infection as measured by p24 levels by approximately 50% [9].

**Fig. 2:**
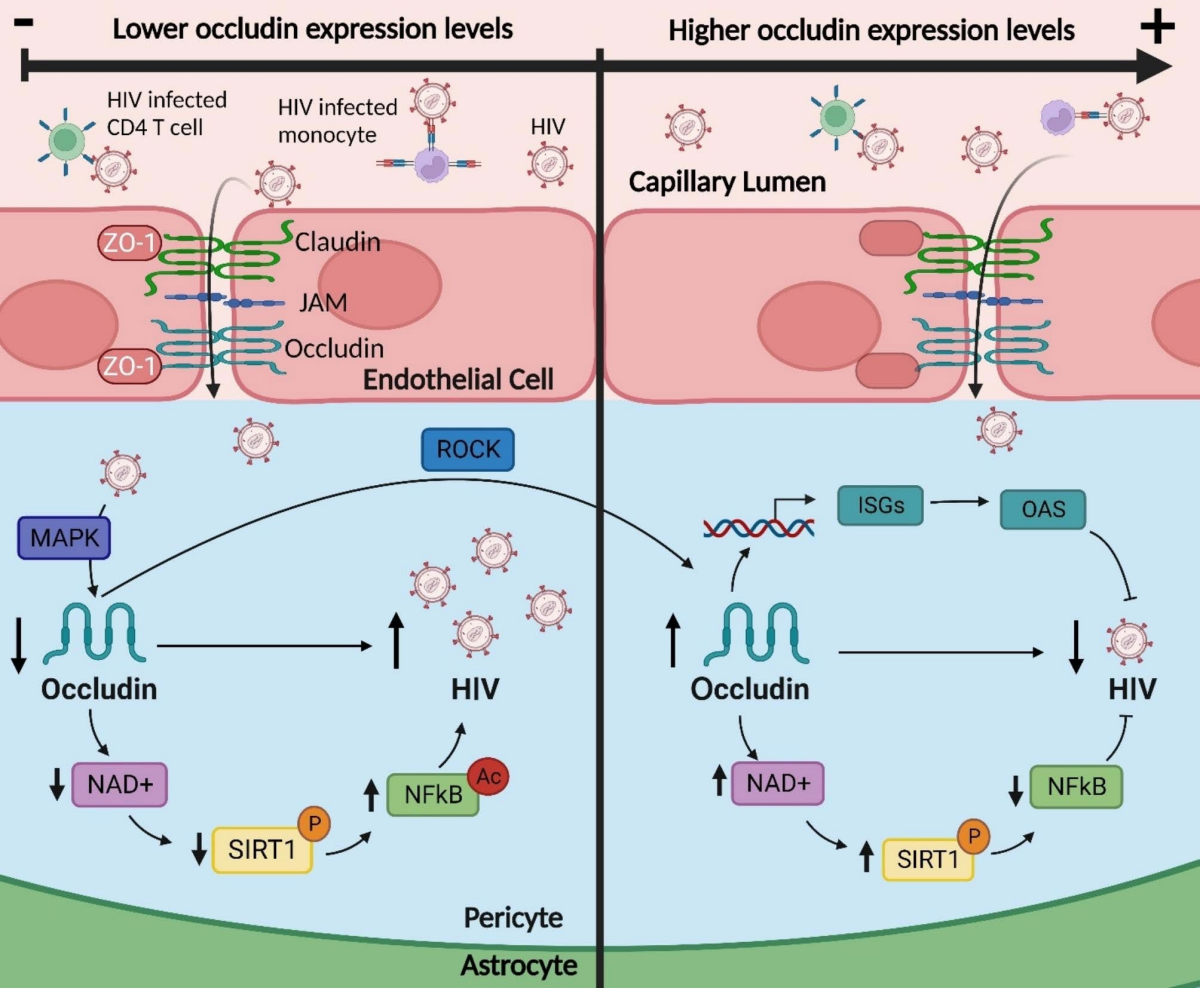
**HIV-1 infection in human brain pericytes under low (left panel) and high (right panel) occludin levels.** A decrease in occludin leads to NAD + depletion, decreasing SIRT1 phosphorylation and increasing NF-κB acetylation, which leads to an increase in HIV-1 replication. In contrast, elevated occludin levels act as an HIV-1 inhibitor by increasing NAD+, following with an increase in phosphorylation of SIRT1 and a decrease in NF-κB acetylation. Moreover, elevated occludin levels increase the expression of interferon-stimulates genes (ISGs) such as the antiviral OAS gene family which degrades viral RNA and provides antiviral protection

The findings reported above suggest that occludin may be a potential target for preventative and pharmacological intervention aimed to eliminate viral reservoirs in PLH. In addition, this data raises the prospect that patients with inflammatory diseases with lower occludin expression may be more susceptible to HIV-1 infection. However, the mechanisms involved in the regulatory interaction between occludin and HIV-1 infection remain largely unknown. At present, two complementary molecular pathways of occludin regulating HIV-1 infection have been identified in human brain pericytes. They include (a) the regulation of the SIRT1 expression by modulation of NAD + and (b) modulation of the antiviral 2′-5′-oligoadenylate synthetase (OAS) gene family through STAT-1 signaling pathway [14, 56, 57] **(**Fig. 2**)**.

### Occludin regulation of HIV-1 Infection through the SIRT1 pathway

SIRT1 is a highly conserved, nicotinamide adenine dinucleotide-dependent class III histone deacetylase [58]. The SIRT1 enzyme deacetylates histone proteins at H3K9, H3K14, H4K16 [59], and H1K26 [60] to control chromatin formation [61]. SIRT1 also deacetylates other proteins [62], with the subsequent modulation of their activity [63]. Examples of such proteins include p53 [62], the RelA/p65 subunit of nuclear factor kappa-light-chain enhancer of activated B cells (NF-κB) [61], forkhead box O transcription factors O4 (FOXO4) [64], O3 (FOXO3) [59], O1 (FOXO1) [65], peroxisome proliferator-activated receptor-γ coactivator-1 alpha (PGC-1α) [66], PGC-1β [67], BMAL1:CLOCK heterodimer [59], and endothelial NOS (eNOS) [68]. SIRT1 also regulates genes related to mitochondrial uncoupling protein 2 (UCP2) [69]. SIRT1 activity decreases PI3K-AKT signaling [70], while increasing AKT-glycogen synthase 3 (GSK3) signaling pathway [71]. Interestingly, SIRT1 can induce AKT activity, while AKT activity may be inhibited by SIRT3/6 [72]. Finally, SIRT1 represses peroxisome proliferator-activated coreceptor gamma (PPAR-γ) transcription [73, 74] and regulates androgen and estrogen receptor responses [75].

SIRT-1 works in concert with AMP-activated protein kinase (AMPK), and it has been reported that occludin levels promote AMPK expression and activation in human pericytes [56]. A study from our laboratory on human brain pericytes showed that occludin, acting as a NADH oxidase, can regulate SIRT1 activity, which influences HIV-1 transcription [14]. Specifically, we identified that occludin has a putative NADH binding site in a pocket formed by complementation of the CC-domain binding site, and can convert NADH to NAD^+^. This process is enhanced upon occludin overexpression and its importance stems from the fact that NAD^+^ is a cofactor regulating SIRT1 activity, which can deacetylate (and thus inactivate) NF-κB, an important stimulator of HIV-1 transcription (Fig. 2). The opposite processes occur when occludin is depleted, which results in a decrease in NAD + levels, decreased p-Ser^47^ phosphorylation of SIRT1, diminished SIRT1 activity, and enhanced activity of NF-κB, which drives HIV-1 transcription [14] (Fig. 2). Indeed, phosphorylation of Ser^27^ emerged as central to SIRT1-based activation mechanisms [76], and also correlated with AMPK activation [77]. In support of the described mechanisms, it was reported that HIV-1 Tat protein can decrease NAD + levels, leading to the deactivation of SIRT1 and the activation of p53 [78]. Moreover, SIRT1 downregulation was linked to increased astrocyte NF-κB activation through Tat upregulation of microRNAs miR-34a and miR-138 [79] and increased levels of SIRT1 functioned as an inhibitor to Tat by upregulating AMPK [80]. Interestingly, higher SIRT1 levels were detected after treatment with anti-HIV integrase transfer inhibitors [81]. It was also demonstrated that the SIRT1 inhibition leads to inflammatory responses in T cells via hyperactivation of NF-κB [82]. SIRT1 can reduce the expression of occludin by impairing the recovery of occludin expression in human brain pericytes [14]. Indeed, SIRT1 overexpression was consistently found to negatively regulate occludin expression in several cell types [83, 84].

### Occludin modulation of HIV-1 replication through STAT-1 molecular pathway

Recently, another study has identified a novel regulatory pathway involving occludin as a regulator of the STAT-1 pathway. The Janus kinase/signal transducer and activator of transcription (JAK/STAT) signaling pathway is a membrane-nucleus signaling involved in the transduction of antiviral genes such as the OAS gene family [85]. After being phosphorylated by the receptor-associated kinases, the STAT family members assemble into homo- or heterodimers that translocate to the nucleus [86]. In the context of HIV-1 infection, STAT-1 was shown to regulate HIV-1 promoter activity and was implicated in the immunopathogenesis of HIV-1 infection and its inflammatory responses [87–90]. Studies have reported that the JAK/STAT signaling pathway could be inhibited by HIV-1 viral gene products, which involve Vif, Vpu, Nef, and Tat, in order to avoid the immune system; however, HIV-1 infection increases STAT-1 expression and overall phosphorylation [88, 91–94].

We reported that occludin levels directly influence the expression of the antiviral interferon stimulated genes (ISGs), such as the OAS genes in human brain pericytes, by regulation of the JAK/STAT-1 molecular pathway. Indeed, overexpression of occludin markedly elevated mRNA levels of ISGs genes such as OAS1, OAS2, OAS3 and OASL, with subsequent protein upregulation and a decrease in HIV replication. Moreover, silencing of occludin (but not silencing of ZO-1) induced an opposing impact, highlighting the importance of occludin in the innate immune regulation to provide antiviral protection [57].

### Occludin phosphorylation as a possible target for HIV-1 Infection in the brain

The occludin function as a BBB structural protein is regulated by phosphorylation processes [95]. Because altering occludin phosphorylation can trigger TJ assembly or disassembly, there is likely a delicate balance between kinase and phosphatase activities acting on occludin. Several protein kinases (PKs) were shown to alter the occludin phosphorylation. Specifically, serine, threonine, and tyrosine occludin residues have been recognized as phosphorylation sites for these kinases [96, 97]. Table 1 summarizes the locations of phosphorylation sites and the kinases that have been identified to modify occludin phosphorylation status. Interestingly, the modification of occludin phosphorylation determines its dimerization and membrane location [98].

**Table 1:** Occludin phosphorylation sites

Kinase	Phosphorylation Site	Model	Mutational analysis	Physiological changes	References
**c-Src**	Tyr ^398^, Tyr^402^,	Rat-1, MDCK (Cell culture	Yes	Regulation of ZO-1, -2, -3.	[99–101]
	Tyr^473^	MDCK (Cell culture)	No	p85α recruitment.	[102]
**CK2**	Thr^400^, Thr^404^, Ser^408^	Caco-2, MDCK (Cell culture)	Yes	CK2-mediated barrier regulation. Regulation of ZO-2.	[103] [104]
	Thr^375^, Ser^379^	X*enopus laevis*	Yes		[105]
**cPKC**	Ser^338^	MDCK (Cell culture)	No		[106]
	Ser^490^	BREC (in vivo)	No	Inhibits TJ trafficking	[107]
**nPKCη**	Thr^403^, Thr^404^, Thr^438^	Caco-2, MDCK (Cell culture)	Yes	Delays assembly at the TJs	[108, 109]
**aPKCζ**	Thr^424^, Thr^438^, Thr^403^, Thr^404^	Rat-1, MDCK, Caco-2 (Cell culture)	Yes	Delays assembly at the TJs	[110] [108]
**ROCK**	Thr^382^, Ser^507^	COS-7 (Cell culture), BMEC (in vivo)	No		[111]
**VEGF**	Ser^490^	BREC (Cell culture)	Yes	Inhibits TJ trafficking	[112]

Abbreviations. Ser, serine; Thr, threonine; Tyr, tyrosine; c-Src, cellular tyrosine-protein kinase Src; CK2, casein protein kinase 2; cPKC, conventional protein kinase C; nPKC, novel protein kinase C; aPKC, atypical protein kinase C; VEGF, vascular endothelial growth factor; ROCK, Rho-associated protein kinase; MDCK, Madin Darby canine kidney cells; Caco-2, human colorectal epithelial adenocarcinoma cells; T84, human colon carcinoma cells; BMEC, brain microvascular endothelial cell; BREC, regulatory B cell; COS-7, African green monkey kidney cells (SV40 transformed)

#### Src kinases in occludin phosphorylation and HIV-1 Infection

**Fig. 3:**
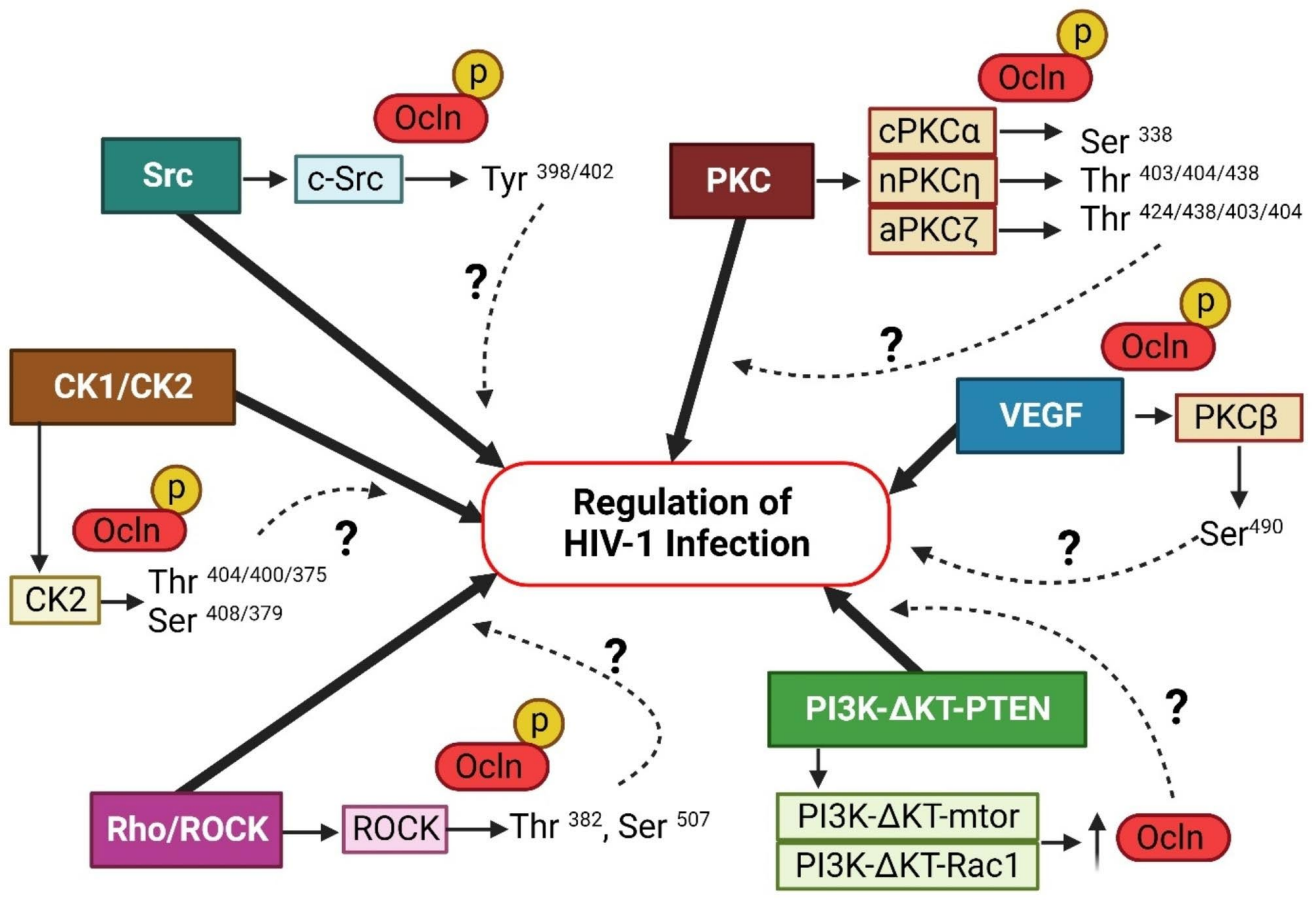
**Proposed model of signaling pathways influencing HIV-1 infection by modulation of occludin expression and function.** Occludin functions are regulated by phosphorylation processes. Src, PKC, Rho-ROCK, VEGF, and PI3K-AKT-PTEN kinases can both alter occludin phosphorylation status and influence HIV-1 infection in several cell types. We propose that occludin phosphorylation may serve as one of the targets to modulate HIV-1 infection

Src kinases are known regulators of occludin phosphorylation (Fig. 3) [28]. For example, c-Src was shown to phosphorylate tyrosine^398/402,473^ on the C-terminus of occludin [99, 101, 102]. Another member of this family of Src kinases is c-Yes, which can also phosphorylate occludin. c-Yes inhibition can lead to occludin redistribution in the cell membranes and altered membrane permeability [113, 114]. Interestingly, some studies reported that c-Src-kinase can regulate HIV-1 infection of immature monocyte-derived dendritic cells [115]. In contrast, other studies have suggested that c-Src activation in HIV-1 infection can prevent early CD4 T-cells infection [116]. An association between the extracellular vesicle-associated c-Src and an increase in latent HIV-1 activation via the PI3K/AKT/mTOR pathway in monocytes and T cells was recently reported [117].

#### PKC kinases in occludin phosphorylation and HIV-1 Infection

Various PKC isoforms can participate in occludin phosphorylation (Fig. 3) [118, 119]. Occludin Ser^338^ has been identified as a potential phosphorylation site for these kinases [106, 120]. PKCα, which can phosphorylate this residue, can also regulate occludin expression [121–123]. In addition, PKCβ activation can also induce occludin phosphorylation [124]. Of the novel PKCs, PKCη targets Thr^403,404,438^ occludin residues. Phosphorylation of more of these target residues increases the presence of occludin in cells membranes [108, 109, 125]. Furthermore, PKCε-mediated phosphorylation can dissociate the ZO-1-occludin complex, thereby disrupting TJ complexes [126]. Atypical PKCζ has also been associated with phosphorylation of occludin and its subsequent reorganization. PKCζ is believed to phosphorylate occludin Thr^403,404,424,438^ residues [108, 110, 127–129].

Interestingly, PKC modulators were being studied to eliminate HIV-infected cells by reactivating latent HIV-1, and then destroying it in an approach named “shock and kill”. Unfortunately, these approaches were not fully successful. While PKC agonists can function as latency-reversing agents to reactivate the virus, they increase cellular resilience to apoptosis [130]. Furthermore, modulators of PKC activation, such as phorbol myristate acetate, can result in the nuclear translocation of NF-κB and enhanced HIV-1 transcription via activation of the HIV-1 long terminal repeat (LTR) [131, 132].

#### Rho and ROCK signaling in occludin phosphorylation and HIV-1 Infection

The Rho–ROCK signaling pathway is formed by Rho GTPase and its downstream effector, Rho-associated kinase (ROCK). The ROCK family contains two isoforms, ROCK1 and ROCK2. The Rho GTPase family contains three subfamilies, Rho (RhoA, RhoB, and RhoC), Rac (Rac1, Rac2, and Rac3), and cell division cycle 42 (Cdc42). Active GTPases are bound to GTP and associated with cell proliferation [133]. Several studies have shown the critical role of the Rho–ROCK pathway in modulating TJs [134–138], including phosphorylation of occludin (Fig. 3) [139]. In mice, occludin residues Thr^382^ and Ser^507^ were the target sites of GST–ROCK [111]. Also, ROCK signaling caused re-localization and regulation [140] [141] of occludin, which suggests that inhibiting the RhoA–ROCK2 pathway can reverse occludin downregulation [141]. However, other investigators indicated that inhibition of ROCK can upregulate occludin [142]. Nevertheless, different ROCK1 and ROCK2 isoforms, as well as different experimental models, may account for this discrepancy.

Interestingly it has been demonstrated that inhibition of Rho-ROCK played a protective role in the BBB maintenance by limiting occludin downregulation in endothelial cells after HIV-1 Tat-treatment [52]. Recently, a potential role of Rho/ROCK in Tat-induced occludin dysregulation, among other TJ proteins, has been shown in C57BL/6 mouse brains [143].

#### PI3K-AKT-PTEN signaling in occludin phosphorylation and HIV-1 Infection

The PI3K–AKT pathway is involved in the regulation of several cell functions, such as cell survival, growth, proliferation, motility, metabolism, angiogenesis, and immune responses [144]. This regulation is, in part, facilitated by AKT-mediated activation of the protein kinase complex, which is the mammalian target of rapamycin (mTOR) [145, 146]; moreover, AKT1, AKT2, and AKT3 have unique functions in cell growth [147].

PI3K is a family of lipid kinases capable of phosphorylating the inositol ring 3′-OH group in inositol phospholipids. Class I PI3Ks are heterodimers formed by a catalytic subunit (p100) and a regulatory subunit (p85) that together catalyze the phosphorylation of phosphatidylinositol 4,5-bisphosphate (PIP3) to phosphatidylinositol 3,4,5-triphosphate (PIP_3_) [148, 149]. PIP_3_ promotes the translocation of the serine/threonine protein kinase AKT to the cell membrane, where it is activated by phosphorylation at the Thr^308^ site by phosphoinositide-dependent kinase 1 (PDK1), while PDK2 phosphorylates the Ser^437^ site [150, 151]. PDK1 is also activated by PIP_3_ since PDK2 is part of the mTORC2 complex [152]. PIP3 can be dephosphorylated by phosphatase and tensin homolog (PTEN), which inhibits AKT activity [153–155]. Meanwhile, AKT can also be directly dephosphorylated at Thr^308^ by protein phosphatase 2 (PP2A) [156] and at Ser^473^ by the PH domain and leucine-rich-repeat-containing protein phosphatases 1 and 2 (PHLPP1/2) [157].

Active AKT is involved in regulating many downstream targets, including mTOR, p21 proteins, Bad, caspase-9, the Wnt–β-catenin pathways, a p53 inhibitor, glycogen synthase kinase (GSK-3β), mouse double minute 2 homolog (MDM2), NF-κB, FOXOs, and cyclic adenosine monophosphate responsive element binding protein 1 (CREB-1) [158–164]. The PI3K-AKT pathway has been described as being involved in TJ alterations [165, 166], and the PI3K regulatory p85 subunit can, among other targets, directly bind to the C-terminus of occludin [167, 168].

The p85 subunit of PI3K is involved in several functions, such as PTEN regulation [169] and phosphorylation of p70S6K [170]; however, it largely serves to regulate PI3K activity. Specifically, phosphorylation of the p85 subunit can enhance PI3K signaling [171]. In contrast, p85 may have primarily inhibitory effects on PI3K signaling in hepatocytes [172]. Binding of PI3K to occludin during oxidative stress was shown to reduce transepithelial electrical resistance (TEER) [168]. However, whether PI3K can directly phosphorylate occludin and the identity of the amino acid targets involved in this phosphorylation both remain unknown. The PI3K–AKT–mTOR pathway of cascading phosphorylation may lead to enhanced occludin production [173], and it has been shown that inducers of this pathway (e.g., celastrol) [174] can prompt occludin expression. It was demonstrated that activation of the PI3K-AKT-Rac1 pathway with acidic fibroblast growth factor (aFGF) can upregulate occludin [175]. Likewise, basic FGF (bFGF) can enhance occludin expression by activating the downstream signaling pathway PI3K-AKT-Rac-1 [176]. In addition, inhibition of PI3K-AKT activity by LY294002 suppressed occludin expression in response to anticancer drugs [177], ferulic acid [178], and/or resveratrol [179]. In human brain microvascular endothelial cells (HBMEC), PI3K inhibition was shown to negate occludin upregulation after silent information regulator 5 (SIRT5) silencing [180], suggesting that PI3K upregulation of occludin may be dependent upon SIRT5 deacetylase activity.

Activation of the PI3K-Akt pathway was demonstrated to induce HIV-1 transcription by activating latent HIV-1 in monocytes and T cells [181]. In addition, PI3K-Akt can prevent the formation of latent HIV-1 reservoirs. As such, PI3K-Akt inhibitors and subsequent downregulation of the PTEN protein resulted in the death of HIV-infected macrophages [182]. Moreover, exposure to HIV-1 Tat protein leads to increased inflammatory cytokine production through the PI3K/Akt and ERK1/2 pathways in astrocytes [183]. Finally, a cross-talk between STAT1 and PI3K-Akt can result in BBB dysfunction in human brain microvascular endothelial cells (Fig. 3) [184].

#### Occludin expression and HIV-1 Infection in response to stimulation by vascular endothelial growth factor (VEGF) and the cell cycle regulators

Vascular endothelial growth factor (VEGF) is an angiogenic factor that was shown to induce phosphorylation and downregulation of occludin [119, 185, 186]. The Ser^490^ occludin residue is the downstream phosphorylation site responsible for inducing ubiquitination of this protein [107, 112, 187, 188]. Interestingly, VEGF can lead to PKCβ activation, with target Ser^490^ occludin phosphorylation. Indeed, it has been shown that inhibition of VEGF blocks occludin Ser^490^ phosphorylation downstream of PKCβ activation [107] (Fig. 3). Additionally, occludin Ser^490^ phosphorylation was demonstrated to be associated with mitotic entry, in which occludin facilitates the process and increases cell proliferation [189]. Recently, research has also suggested that activating the VEGF-Flk-1-ERK pathway causes occludin tyrosine phosphorylation [190].

VEGF has been characterized to have neuroprotective effects and was present at higher levels during neurocognitive disorders [191, 192]. This was possibly due to its role in maintaining proper functions of neurons and glial cells, as well as in blood vessel formation [193]. On the other hand, VEGF is a strong proinflammatory factor. In PLH, T cells have been shown to upregulate VEGF due to inflammatory signals [194]. An inverse relationship was observed between blood VEGF-D concentrations and amnestic mild cognitive impairment in older people with HIV-1 [191]. In patients with HIV encephalopathy, the serum concentration of VEGF was increased in comparison to PLH without this comorbidity [192]. In addition, the HIV-1 Tat protein was found to damage microvessels and reduce VEGF levels, suggesting a possible role in neurocognitive decline in HIV-1 infection [195].

The casein kinase 1 and 2 (CK1 and CK2) are serine/threonine kinases that, among other proteins, phosphorylate occludin. There may be multiple regulatory regions on occludin that are affected by CK1-ε; at the same time, the C-terminal region of occludin can inhibit CK1-ε phosphorylation [196]. However, much more is known about the involvement of CK2 in occludin phosphorylation. CK2 phosphorylates three amino acid residues- Thr^404^, Ser^408^, and Thr^400^ - in the human occludin C-terminus. Thr^375^ and Ser^379^ have also been described in *Xenopus laevis* as potential CK2 phosphorylation sites [103–105, 197]. Also, inhibiting CK2 leads to overexpression of occludin [103]. Several papers have described a link between CK2 and HIV-1 replication proteins [198]. While the substrate of this interaction remains unknown, it has been shown that HIV-1 Rev can activate CK2, which then can induce HIV-1 Rev phosphorylation [199]. Moreover, it also has been described that multiple HIV-1 gene products can be phosphorylated by CK2 [198, 199].

### Concluding remarks and future perspectives

Occludin plays a key role in maintaining the integrity and permeability of the BBB [200] and it has been reported that modification or loss of occludin expression levels are associated with increase neurological damage in several diseases such as ischemic stroke [201, 202] or status epilepticus [203]. A recent study has also shown an increase in serum zonulin and ocln levels in people with bipolar disorder [204] and children with autism spectrum disorder [205]. Importantly, structure and function alterations of the BBB are characteristics hallmarks in brains infected by HIV-1. In fact, alterations in occludin expression levels have been associated with BBB damage during HIV infection.

Traditionally, occludin has been considered a multifunctional TJ protein regulating endothelial and epithelial structure and function. However, occludin is ubiquitously expressed in several cells and tissues, which suggests much broader functions than those assigned to regulating the integrity of tissue barriers. In fact, although is mainly known for its role as a TJ, occludin protein is also a multifunctional protein that can influence cellular metabolism acting as a NADH oxidase.

Recently, occludin has attracted importance due to its newly discovered metabolic functions and its role in controlling HIV-1 infection. Various mechanistic pathways have been proposed to be involved in this effect, such as regulating the expression of the OAS gene family through the STAT-1 signaling pathway, or by regulating the SIRT1 activity through NAD+. Occludin functions appear to be influenced by its phosphorylation, and several signaling pathways can be involved in this process including Src, PKC, CK2, Rho-ROCK, VEGF, and PI3K-AKT-PTEN **(**Table 1**).** Importantly, the kinases involved in occludin phosphorylation can also influence HIV-1 infection **(**Fig. 3**)**. The present review describes the cross-talks between phosphorylation of occludin and regulation of HIV-1 infection; however, it is not fully understood whether these are unrelated or causative associations. Unfortunately, no preclinical study has focused on occludin as a possible target in HIV-1 infection. We propose that a better understanding of the occludin-HIV-1 infections may identify occludin as a possible target to control HIV-1 infection and improve the life of people living with HIV-1.

## Data Availability

Available upon request.

## References

[1] Ismael S, Moshahid Khan M, Kumar P, Kodidela S, Mirzahosseini G, Kumar S et al. HIV Associated Risk factors for ischemic Stroke and future perspectives. Int J Mol Sci. 2020;21(15). 10.3390/ijms21155306 PMC7432359 32722629

[2] Cohen MS, Chen YQ, McCauley M, Gamble T, Hosseinipour MC, Kumarasamy N (2011). Prevention of HIV-1 Infection with early antiretroviral therapy. N Engl J Med.

[3] Cohen MS, Chen YQ, McCauley M, Gamble T, Hosseinipour MC, Kumarasamy N (2016). Antiretroviral therapy for the Prevention of HIV-1 transmission. N Engl J Med.

[4] Toborek M, Lee YW, Flora G, Pu H, Andras IE, Wylegala E (2005). Mechanisms of the blood-brain barrier disruption in HIV-1 Infection. Cell Mol Neurobiol.

[5] Narasipura SD, Kim S, Al-Harthi L (2014). Epigenetic regulation of HIV-1 latency in astrocytes. J Virol.

[6] Bertrand L, Cho HJ, Toborek M (2019). Blood-brain barrier pericytes as a target for HIV-1 Infection. Brain.

[7] Bertrand L, Meroth F, Tournebize M, Leda AR, Sun E, Toborek M. Targeting the HIV-infected brain to improve ischemic Stroke outcome. Nat Commun. 2019;10. 10.1038/s41467-019-10046-x PMC6494822 31043599

[8] Wallet C, De Rovere M, Van Assche J, Daouad F, De Wit S, Gautier V (2019). Microglial cells: the Main HIV-1 Reservoir in the brain. Front Cell Infect Microbiol.

[9] Torices S, Roberts SA, Park M, Malhotra A, Toborek M, Occludin (2020). caveolin-1, and Alix form a multi-protein complex and regulate HIV-1 Infection of brain pericytes. FASEB Journal: Official Publication of the Federation of American Societies for Experimental Biology.

[10] Torices S, Cabrera R, Stangis M, Naranjo O, Fattakhov N, Teglas T (2021). Expression of SARS-CoV-2-related receptors in cells of the neurovascular unit: implications for HIV-1 Infection. J Neuroinflammation.

[11] Schouten J, Cinque P, Gisslen M, Reiss P, Portegies P (2011). HIV-1 Infection and cognitive impairment in the cART era: a review. AIDS.

[12] Kranick SM, Nath A (2012). Neurologic Complications of HIV-1 Infection and its treatment in the era of antiretroviral therapy. Continuum (Minneap Minn).

[13] Daneman R, Prat A (2015). The blood-brain barrier. Cold Spring Harb Perspect Biol.

[14] Castro V, Bertrand L, Luethen M, Dabrowski S, Lombardi J, Morgan L (2016). Occludin controls HIV transcription in brain pericytes via regulation of SIRT-1 activation. FASEB J.

[15] Andersson LM, Hagberg L, Fuchs D, Svennerholm B, Gisslen M (2001). Increased blood-brain barrier permeability in neuro-asymptomatic HIV-1-infected individuals–correlation with cerebrospinal fluid HIV-1 RNA and neopterin levels. J Neurovirol.

[16] Feldman GJ, Mullin JM, Ryan MP (2005). Occludin: structure, function and regulation. Adv Drug Deliv Rev.

[17] Furuse M, Hirase T, Itoh M, Nagafuchi A, Yonemura S, Tsukita S (1993). Occludin: a novel integral membrane protein localizing at tight junctions. J Cell Biol.

[18] Yaffe Y, Shepshelovitch J, Nevo-Yassaf I, Yeheskel A, Shmerling H, Kwiatek JM (2012). The MARVEL transmembrane motif of occludin mediates oligomerization and targeting to the basolateral surface in epithelia. J Cell Sci.

[19] Wang S, Li Y, Han F, Hu J, Yue L, Yu Y (2009). Identification and characterization of MARVELD1, a novel nuclear protein that is down-regulated in multiple cancers and silenced by DNA methylation. Cancer Lett.

[20] Raleigh DR, Marchiando AM, Zhang Y, Shen L, Sasaki H, Wang Y (2010). Tight junction-associated MARVEL proteins marveld3, tricellulin, and occludin have distinct but overlapping functions. Mol Biol Cell.

[21] Cummins PM (2012). Occludin: one protein, many forms. Mol Cell Biol.

[22] Steed E, Rodrigues NT, Balda MS, Matter K (2009). Identification of MarvelD3 as a tight junction-associated transmembrane protein of the occludin family. BMC Cell Biol.

[23] Saitou M, Furuse M, Sasaki H, Schulzke JD, Fromm M, Takano H (2000). Complex phenotype of mice lacking occludin, a component of tight junction strands. Mol Biol Cell.

[24] Li Y, Fanning AS, Anderson JM, Lavie A (2005). Structure of the conserved cytoplasmic C-terminal domain of occludin: identification of the ZO-1 binding surface. J Mol Biol.

[25] Muresan Z, Paul DL, Goodenough DA (2000). Occludin 1B, a variant of the tight Junction protein occludin. Mol Biol Cell.

[26] Balda MS, Matter K (2000). Transmembrane proteins of tight junctions. Semin Cell Dev Biol.

[27] Traweger A, Fang D, Liu YC, Stelzhammer W, Krizbai IA, Fresser F (2002). The tight junction-specific protein occludin is a functional target of the E3 ubiquitin-protein ligase itch. J Biol Chem.

[28] Dorfel MJ, Huber O (2012). Modulation of tight junction structure and function by kinases and phosphatases targeting occludin. J Biomed Biotechnol.

[29] Bamforth SD, Kniesel U, Wolburg H, Engelhardt B, Risau W (1999). A dominant mutant of occludin disrupts tight junction structure and function. J Cell Sci.

[30] Chen Y, Merzdorf C, Paul DL, Goodenough DA (1997). COOH terminus of occludin is required for tight junction barrier function in early Xenopus embryos. J Cell Biol.

[31] Dorfel MJ, Westphal JK, Huber O (2009). Differential phosphorylation of occludin and tricellulin by CK2 and CK1. Ann N Y Acad Sci.

[32] Hwang I, An BS, Yang H, Kang HS, Jung EM, Jeung EB (2013). Tissue-specific expression of occludin, zona occludens-1, and junction adhesion molecule A in the duodenum, ileum, colon, kidney, liver, lung, brain, and skeletal muscle of C57BL mice. J Physiol Pharmacol.

[33] Romanitan MO, Popescu BO, Winblad B, Bajenaru OA, Bogdanovic N (2007). Occludin is overexpressed in Alzheimer’s Disease and vascular Dementia. J Cell Mol Med.

[34] Wachtel M, Bolliger MF, Ishihara H, Frei K, Bluethmann H, Gloor SM (2001). Down-regulation of occludin expression in astrocytes by tumour necrosis factor (TNF) is mediated via TNF type-1 receptor and nuclear factor-κB activation. J Neurochem.

[35] Ni Y, Teng T, Li R, Simonyi A, Sun GY, Lee JC (2017). TNFα alters occludin and cerebral endothelial permeability: role of p38MAPK. PLoS ONE.

[36] Murata M, Kojima T, Yamamoto T, Go M, Takano K-i, Osanai M (2005). Down-regulation of survival signaling through MAPK and Akt in occludin-deficient mouse hepatocytes in vitro. Exp Cell Res.

[37] Alexander J, Dayton T, Davis C, Hill S, Jackson T, Blaschuk O (1999). Activated T-Lymphocytes Express Occludin, a component of tight junctions. Inflammation.

[38] Watters AK, Rom S, Hill JD, Dematatis MK, Zhou Y, Merkel SF (2015). Identification and dynamic regulation of tight junction protein expression in human neural stem cells. Stem Cells Dev.

[39] Ronaldson PT, Davis TP (2020). Regulation of blood-brain barrier integrity by microglia in health and Disease: a therapeutic opportunity. J Cereb Blood Flow Metab.

[40] Mehrabadi AR, Korolainen MA, Odero G, Miller DW, Kauppinen TM (2017). Poly(ADP-ribose) polymerase-1 regulates microglia mediated decrease of endothelial tight junction integrity. Neurochem Int.

[41] Ashwood P, Harvey R, Verjee T, Wolstencroft R, Thompson RP, Powell JJ (2004). Functional interactions between mucosal IL-1, IL-ra and TGF-beta 1 in ulcerative Colitis. Inflamm Res.

[42] Bauer H, Stelzhammer W, Fuchs R, Weiger TM, Danninger C, Probst G (1999). Astrocytes and neurons express the tight junction-specific protein occludin in vitro. Exp Cell Res.

[43] Cho HJ, Kuo AM, Bertrand L, Toborek M (2017). HIV alters Gap Junction-mediated intercellular communication in human brain pericytes. Front Mol Neurosci.

[44] Kaur G, Pant P, Bhagat R, Seth P (2023). Zika virus E protein modulates functions of human brain microvascular endothelial cells and astrocytes: implications on blood-brain barrier properties. Front Cell Neurosci.

[45] Afonso PV, Ozden S, Cumont MC, Seilhean D, Cartier L, Rezaie P (2008). Alteration of blood-brain barrier integrity by retroviral Infection. PLoS Pathog.

[46] Gralinski LE, Ashley SL, Dixon SD, Spindler KR (2009). Mouse adenovirus type 1-induced breakdown of the blood-brain barrier. J Virol.

[47] Vaswani CM, Varkouhi AK, Gupta S, Ektesabi AM, Tsoporis JN, Yousef S (2023). Preventing occludin tight-junction disruption via inhibition of microRNA-193b-5p attenuates viral load and influenza-induced lung injury. Mol Ther.

[48] Mailly L, Baumert TF (2020). Hepatitis C virus Infection and tight junction proteins: the ties that bind. Biochim Biophys Acta Biomembr.

[49] Ruan T, Sun J, Liu W, Prinz RA, Peng D, Liu X (2020). H1N1 Influenza virus cross-activates Gli1 to disrupt the intercellular junctions of alveolar epithelial cells. Cell Rep.

[50] Ploss A, Evans MJ, Gaysinskaya VA, Panis M, You H, de Jong YP (2009). Human occludin is a Hepatitis C virus entry factor required for Infection of mouse cells. Nature.

[51] Rice AP (2017). The HIV-1 Tat protein: mechanism of Action and Target for HIV-1 cure strategies. Curr Pharm Des.

[52] Chen Y, Huang W, Jiang W, Wu X, Ye B, Zhou X (2016). HIV-1 Tat regulates occludin and abeta transfer receptor expression in Brain endothelial cells via Rho/ROCK signaling pathway. Oxid Med Cell Longev.

[53] Xu R, Feng X, Xie X, Zhang J, Wu D, Xu L (2012). HIV-1 Tat protein increases the permeability of brain endothelial cells by both inhibiting occludin expression and cleaving occludin via matrix metalloproteinase-9. Brain Res.

[54] Lassiter C, Fan X, Joshi PC, Jacob BA, Sutliff RL, Jones DP (2009). HIV-1 transgene expression in rats causes oxidant stress and alveolar epithelial barrier dysfunction. AIDS Res Ther.

[55] Ohene-Nyako M, Persons AL, Napier TC (2021). Hippocampal blood-brain barrier of methamphetamine self-administering HIV-1 transgenic rats. Eur J Neurosci.

[56] Castro V, Skowronska M, Lombardi J, He J, Seth N, Velichkovska M (2018). Occludin regulates glucose uptake and ATP production in pericytes by influencing AMP-activated protein kinase activity. J Cereb Blood Flow Metab.

[57] Torices S, Teglas T, Naranjo O, Fattakhov N, Frydlova K, Cabrera R et al. Occludin regulates HIV-1 Infection by modulation of the Interferon Stimulated OAS Gene Family. Mol Neurobiol. 2023:1–17. 10.1007/s12035-023-03381-0 PMC10199280 37209263

[58] Kalous KS, Wynia-Smith SL, Olp MD, Smith BC (2016). Mechanism of Sirt1 NAD+-dependent protein deacetylase inhibition by Cysteine S-Nitrosation. J Biol Chem.

[59] Nakahata Y, Kaluzova M, Grimaldi B, Sahar S, Hirayama J, Chen D (2008). The NAD+-dependent deacetylase SIRT1 modulates CLOCK-mediated chromatin remodeling and circadian control. Cell.

[60] Vaquero A, Scher M, Lee D, Erdjument-Bromage H, Tempst P, Reinberg D (2004). Human SirT1 interacts with histone H1 and promotes formation of facultative heterochromatin. Mol Cell.

[61] Chen GD, Yu WD, Chen XP (2016). SirT1 activator represses the transcription of TNFalpha in THP1 cells of a sepsis model via deacetylation of H4K16. Mol Med Rep.

[62] Stunkel W, Peh BK, Tan YC, Nayagam VM, Wang X, Salto-Tellez M (2007). Function of the SIRT1 protein deacetylase in cancer. Biotechnol J.

[63] Canto C, Auwerx J (2009). PGC-1alpha, SIRT1 and AMPK, an energy sensing network that controls energy expenditure. Curr Opin Lipidol.

[64] Kobayashi Y, Furukawa-Hibi Y, Chen C, Horio Y, Isobe K, Ikeda K (2005). SIRT1 is critical regulator of FOXO-mediated transcription in response to oxidative stress. Int J Mol Med.

[65] Frescas D, Valenti L, Accili D (2005). Nuclear trapping of the forkhead transcription factor FoxO1 via sirt-dependent deacetylation promotes expression of glucogenetic genes. J Biol Chem.

[66] Zhou Y, Wang S, Li Y, Yu S, Zhao Y (2018). SIRT1/PGC-1α signaling promotes mitochondrial functional recovery and reduces apoptosis after Intracerebral Hemorrhage in rats. Front Mol Neurosci.

[67] Wang R, Li Jing J, Diao S, Kwak Y-D, Liu L, Zhi L (2013). Metabolic stress modulates Alzheimer’s β-Secretase gene transcription via SIRT1-PPARγ-PGC-1 in neurons. Cell Metabol.

[68] Jung S-B, Kwon SK, Kwon M, Nagar H, Jeon BH, Irani K (2013). Docosahexaenoic acid improves vascular function via up-regulation of SIRT1 expression in endothelial cells. Biochem Biophys Res Commun.

[69] Moynihan KA, Grimm AA, Plueger MM, Bernal-Mizrachi E, Ford E, Cras-Meneur C (2005). Increased dosage of mammalian Sir2 in pancreatic beta cells enhances glucose-stimulated insulin secretion in mice. Cell Metab.

[70] Wang H, Liu H, Chen K, Xiao J, He K, Zhang J (2012). SIRT1 promotes tumorigenesis of hepatocellular carcinoma through PI3K/PTEN/AKT signaling. Oncol Rep.

[71] Li XH, Chen C, Tu Y, Sun HT, Zhao ML, Cheng SX (2013). Sirt1 promotes axonogenesis by deacetylation of Akt and inactivation of GSK3. Mol Neurobiol.

[72] Pillai VB, Sundaresan NR, Gupta MP (2014). Regulation of akt signaling by sirtuins: its implication in cardiac hypertrophy and aging. Circul Res.

[73] Picard F, Kurtev M, Chung N, Topark-Ngarm A, Senawong T, de Machado R (2004). Sirt1 promotes fat mobilization in white adipocytes by repressing PPAR-γ. Nature.

[74] Yao H, Yao Z, Zhang S, Zhang W, Zhou W (2018). Upregulation of SIRT1 inhibits H2O2–induced osteoblast apoptosis via FoxO1/β–catenin pathway. Mol Med Rep.

[75] Shoba B, Lwin ZM, Ling LS, Bay B-H, Yip GW, Kumar SD (2009). Function of sirtuins in Biological tissues. Anat Rec.

[76] Wen L, Chen Z, Zhang F, Cui X, Sun W, Geary GG (2013). Ca2+/calmodulin-dependent protein kinase kinase beta phosphorylation of Sirtuin 1 in endothelium is atheroprotective. Proc Natl Acad Sci U S A.

[77] Zhang ZY, Hong D, Nam SH, Kim JM, Paik YH, Joh JW (2015). SIRT1 regulates oncogenesis via a mutant p53-dependent pathway in hepatocellular carcinoma. J Hepatol.

[78] Thakur BK, Chandra A, Dittrich T, Welte K, Chandra P (2012). Inhibition of SIRT1 by HIV-1 viral protein Tat results in activation of p53 pathway. Biochem Biophys Res Commun.

[79] Hu G, Liao K, Yang L, Pendyala G, Kook Y, Fox HS (2017). Tat-mediated induction of miRs-34a & -138 promotes astrocytic activation via downregulation of SIRT1: implications for aging in HAND. J Neuroimmune Pharmacol.

[80] Kim MJ, Ahn K, Park SH, Kang HJ, Jang BG, Oh SJ (2009). SIRT1 regulates tyrosine hydroxylase expression and differentiation of neuroblastoma cells via FOXO3a. FEBS Lett.

[81] Jurkowska K, Szymanska B, Knysz B, Piwowar A. The effect of antiretroviral therapy on SIRT1, SIRT3 and SIRT6 expression in HIV-Infected patients. Molecules. 2022;27(4). 10.3390/molecules27041358 PMC8879865 35209148

[82] Kwon HS, Brent MM, Getachew R, Jayakumar P, Chen LF, Schnolzer M (2008). Human immunodeficiency virus type 1 Tat protein inhibits the SIRT1 deacetylase and induces T cell hyperactivation. Cell Host Microbe.

[83] Cheng F, Su L, Yao C, Liu L, Shen J, Liu C (2016). SIRT1 promotes epithelial–mesenchymal transition and Metastasis in Colorectal cancer by regulating Fra-1 expression. Cancer Lett.

[84] Cha BK, Kim YS, Hwang KE, Cho KH, Oh SH, Kim BR (2016). Celecoxib and sulindac inhibit TGF-beta1-induced epithelial-mesenchymal transition and suppress Lung cancer migration and invasion via downregulation of sirtuin 1. Oncotarget.

[85] Kotenko SV, Gallagher G, Baurin VV, Lewis-Antes A, Shen M, Shah NK (2003). IFN-lambdas mediate antiviral protection through a distinct class II cytokine receptor complex. Nat Immunol.

[86] Darnell JE (1998). Jr. Studies of IFN-induced transcriptional activation uncover the Jak-Stat pathway. J Interferon Cytokine Res.

[87] Bhargavan B, Woollard SM, Kanmogne GD (2016). Data in support of NFkappaB and JNK pathways involvement in TLR3-mediated HIV-1 transactivation, expression of IL-6 and transcription factors associated with HIV-1 replication. Data Brief.

[88] Bovolenta C, Camorali L, Lorini AL, Ghezzi S, Vicenzi E, Lazzarin A (1999). Constitutive activation of STATs upon in vivo human immunodeficiency virus Infection. Blood.

[89] Cheng J, Myers TG, Levinger C, Kumar P, Kumar J, Goshu BA (2022). IL-27 induces IFN/STAT1-dependent genes and enhances function of TIGIT(+) HIVGag-specific T cells. iScience.

[90] Dupont M, Rousset S, Manh TV, Monard SC, Pingris K, Souriant S (2022). Dysregulation of the IFN-I signaling pathway by Mycobacterium tuberculosis leads to exacerbation of HIV-1 Infection of macrophages. J Leukoc Biol.

[91] Bovolenta C, Pilotti E, Mauri M, Panzeri B, Sassi M, Dall’Aglio P (2002). Retroviral interference on STAT activation in individuals coinfected with human T cell Leukemia virus type 2 and HIV-1. J Immunol.

[92] Gargan S, Ahmed S, Mahony R, Bannan C, Napoletano S, O’Farrelly C (2018). HIV-1 promotes the degradation of components of the type 1 IFN JAK/STAT pathway and blocks anti-viral ISG induction. EBioMedicine.

[93] Li JC, Au KY, Fang JW, Yim HC, Chow KH, Ho PL (2011). HIV-1 trans-activator protein dysregulates IFN-gamma signaling and contributes to the suppression of autophagy induction. AIDS.

[94] Magnani M, Balestra E, Fraternale A, Aquaro S, Paiardini M, Cervasi B (2003). Drug-loaded red blood cell-mediated clearance of HIV-1 macrophage reservoir by selective inhibition of STAT1 expression. J Leukoc Biol.

[95] Cong X, Kong W (2020). Endothelial tight junctions and their regulatory signaling pathways in vascular homeostasis and Disease. Cell Signal.

[96] Farshori P, Kachar B (1999). Redistribution and phosphorylation of occludin during opening and resealing of tight junctions in cultured epithelial cells. J Membr Biol.

[97] Tsukamoto T, Nigam SK (1999). Role of tyrosine phosphorylation in the reassembly of occludin and other tight junction proteins. Am J Physiol.

[98] McCaffrey G, Seelbach MJ, Staatz WD, Nametz N, Quigley C, Campos CR (2008). Occludin oligomeric assembly at tight junctions of the blood-brain barrier is disrupted by peripheral inflammatory hyperalgesia. J Neurochem.

[99] Elias BC, Suzuki T, Seth A, Giorgianni F, Kale G, Shen L (2009). Phosphorylation of Tyr-398 and Tyr-402 in occludin prevents its interaction with ZO-1 and destabilizes its assembly at the tight junctions. J Biol Chem.

[100] Kale G, Naren AP, Sheth P, Rao RK (2003). Tyrosine phosphorylation of occludin attenuates its interactions with ZO-1, ZO-2, and ZO-3. Biochem Biophys Res Commun.

[101] Dorfel MJ, Huber O (2012). A phosphorylation hotspot within the occludin C-terminal domain. Ann N Y Acad Sci.

[102] Du D, Xu F, Yu L, Zhang C, Lu X, Yuan H (2010). The tight junction protein, occludin, regulates the directional migration of epithelial cells. Dev Cell.

[103] Raleigh DR, Boe DM, Yu D, Weber CR, Marchiando AM, Bradford EM (2011). Occludin S408 phosphorylation regulates tight junction protein interactions and barrier function. J Cell Biol.

[104] Dorfel MJ, Westphal JK, Bellmann C, Krug SM, Cording J, Mittag S (2013). CK2-dependent phosphorylation of occludin regulates the interaction with ZO-proteins and tight junction integrity. Cell Commun Signal.

[105] Cordenonsi M, Turco F, D’Atri F, Hammar E, Martinucci G, Meggio F (1999). Xenopus laevis occludin. Identification of in vitro phosphorylation sites by protein kinase CK2 and association with cingulin. Eur J Biochem.

[106] Andreeva AY, Krause E, Muller EC, Blasig IE, Utepbergenov DI (2001). Protein kinase C regulates the phosphorylation and cellular localization of occludin. J Biol Chem.

[107] Murakami T, Frey T, Lin C, Antonetti DA (2012). Protein kinase cbeta phosphorylates occludin regulating tight junction trafficking in vascular endothelial growth factor-induced permeability in vivo. Diabetes.

[108] Suzuki T, Elias BC, Seth A, Shen L, Turner JR, Giorgianni F (2009). PKC eta regulates occludin phosphorylation and epithelial tight junction integrity. Proc Natl Acad Sci U S A.

[109] Andreeva AY, Piontek J, Blasig IE, Utepbergenov DI (2006). Assembly of tight junction is regulated by the antagonism of conventional and novel protein kinase C isoforms. Int J Biochem Cell Biol.

[110] Jain S, Suzuki T, Seth A, Samak G, Rao R (2011). Protein kinase czeta phosphorylates occludin and promotes assembly of epithelial tight junctions. Biochem J.

[111] Yamamoto M, Ramirez SH, Sato S, Kiyota T, Cerny RL, Kaibuchi K (2008). Phosphorylation of claudin-5 and occludin by rho kinase in brain endothelial cells. Am J Pathol.

[112] Murakami T, Felinski EA, Antonetti DA (2009). Occludin phosphorylation and ubiquitination regulate tight junction trafficking and vascular endothelial growth factor-induced permeability. J Biol Chem.

[113] Chen YH, Lu Q (2003). Association of nonreceptor tyrosine kinase c-yes with tight junction protein occludin by coimmunoprecipitation assay. Methods Mol Biol.

[114] Xiao X, Mruk DD, Lee WM, Cheng CY (2011). c-Yes regulates cell adhesion at the blood-testis barrier and the apical ectoplasmic specialization in the seminiferous epithelium of rat testes. Int J Biochem Cell Biol.

[115] Gilbert C, Barat C, Cantin R, Tremblay MJ (2007). Involvement of src and syk tyrosine kinases in HIV-1 transfer from dendritic cells to CD4 + T lymphocytes. J Immunol.

[116] Garner BR, Burrus O, Ortiz A, Tueller SJ, Peinado S, Hedrick H (2022). A longitudinal mixed-methods examination of positive health check: implementation results from a type 1 effectiveness-implementation hybrid trial. J Acquir Immune Defic Syndr.

[117] Barclay RA, Mensah GA, Cowen M, DeMarino C, Kim Y, Pinto DO et al. Extracellular vesicle activation of latent HIV-1 is driven by EV-Associated c-Src and Cellular SRC-1 via the PI3K/AKT/mTOR pathway. Viruses. 2020;12(6). 10.3390/v12060665 PMC7354524 32575590

[118] Cao X, Lin H, Muskhelishvili L, Latendresse J, Richter P, Heflich RH (2015). Tight junction disruption by cadmium in an in vitro human airway tissue model. Respir Res.

[119] Annese T, Ruggieri S, De Giorgis M, Ribatti D, Tamma R, Nico B (2019). Alpha-methyl-prednisolone normalizes the PKC mediated brain angiogenesis in dystrophic mdx mice. Brain Res Bull.

[120] Zhou Y, Qin H, Zhang M, Shen T, Chen H, Ma Y (2010). Lactobacillus plantarum inhibits intestinal epithelial barrier dysfunction induced by unconjugated bilirubin. Br J Nutr.

[121] Yu H, Wang C, Wang X, Wang H, Zhang C, You J (2017). Long-term exposure to ethanol downregulates tight junction proteins through the protein kinase calpha signaling pathway in human cerebral microvascular endothelial cells. Exp Ther Med.

[122] Liu J, Liu L, Chao S, Liu Y, Liu X, Zheng J (2017). The role of miR-330-3p/PKC-alpha signaling pathway in low-dose endothelial-monocyte activating Polypeptide-II increasing the permeability of blood-tumor barrier. Front Cell Neurosci.

[123] Yu H, Wang P, An P, Xue Y (2012). Recombinant human angiopoietin-1 ameliorates the expressions of ZO-1, occludin, VE-cadherin, and PKCalpha signaling after focal cerebral ischemia/reperfusion in rats. J Mol Neurosci.

[124] Srivastava K, Shao B, Bayraktutan U (2013). PKC-beta exacerbates in vitro brain barrier damage in hyperglycemic settings via regulation of RhoA/Rho-kinase/MLC2 pathway. J Cereb Blood Flow Metab.

[125] Nishitsuji K, Hosono T, Nakamura T, Bu G, Michikawa M (2011). Apolipoprotein E regulates the integrity of tight junctions in an isoform-dependent manner in an in vitro blood-brain barrier model. J Biol Chem.

[126] Chattopadhyay R, Dyukova E, Singh NK, Ohba M, Mobley JA, Rao GN (2014). Vascular endothelial tight junctions and barrier function are disrupted by 15(S)-hydroxyeicosatetraenoic acid partly via protein kinase C epsilon-mediated zona occludens-1 phosphorylation at threonine 770/772. J Biol Chem.

[127] Li Z, Liu YH, Liu XB, Xue YX, Wang P, Liu LB (2015). Low-dose endothelial monocyte-activating polypeptide-II increases permeability of blood-tumor barrier via a PKC-zeta/PP2A-dependent signaling mechanism. Exp Cell Res.

[128] Kalsi KK, Garnett JP, Patkee W, Weekes A, Dockrell ME, Baker EH (2019). Metformin attenuates the effect of Staphylococcus aureus on airway tight junctions by increasing PKCzeta-mediated phosphorylation of occludin. J Cell Mol Med.

[129] Willis CL, Meske DS, Davis TP (2010). Protein kinase C activation modulates reversible increase in cortical blood-brain barrier permeability and tight junction protein expression during hypoxia and posthypoxic reoxygenation. J Cereb Blood Flow Metab.

[130] French AJ, Natesampillai S, Krogman A, Correia C, Peterson KL, Alto A (2020). Reactivating latent HIV with PKC agonists induces resistance to apoptosis and is associated with phosphorylation and activation of BCL2. PLoS Pathog.

[131] DeChristopher BA, Loy BA, Marsden MD, Schrier AJ, Zack JA, Wender PA (2012). Designed, synthetically accessible bryostatin analogues potently induce activation of latent HIV reservoirs in vitro. Nat Chem.

[132] Mehla R, Bivalkar-Mehla S, Zhang R, Handy I, Albrecht H, Giri S (2010). Bryostatin modulates latent HIV-1 Infection via PKC and AMPK signaling but inhibits acute Infection in a receptor Independent manner. PLoS ONE.

[133] ZOHRABIAN VM, CHAU FORZANIB, MURALI Z, JHANWAR-UNIYAL R (2009). Rho/ROCK and MAPK signaling pathways are involved in Glioblastoma Cell Migration and Proliferation. Anticancer Res.

[134] Soliman M, Cho EH, Park JG, Kim JY, Alfajaro MM, Baek YB (2018). Rotavirus-Induced Early activation of the RhoA/ROCK/MLC signaling pathway mediates the disruption of tight junctions in polarized MDCK cells. Sci Rep.

[135] Xue Y, He JT, Zhang KK, Chen LJ, Wang Q, Xie XL (2019). Methamphetamine reduces expressions of tight junction proteins, rearranges F-actin cytoskeleton and increases the blood brain barrier permeability via the RhoA/ROCK-dependent pathway. Biochem Biophys Res Commun.

[136] Jou TS, Schneeberger EE, Nelson WJ (1998). Structural and functional regulation of tight junctions by RhoA and Rac1 small GTPases. J Cell Biol.

[137] Bruewer M, Hopkins AM, Hobert ME, Nusrat A, Madara JL, RhoA (2004). Rac1, and Cdc42 exert distinct effects on epithelial barrier via selective structural and biochemical modulation of junctional proteins and F-actin. Am J Physiol Cell Physiol.

[138] Zhong Y, Zhang B, Eum SY, Toborek M (2012). HIV-1 Tat triggers nuclear localization of ZO-1 via rho signaling and cAMP response element-binding protein activation. J Neuroscience: Official J Soc Neurosci.

[139] Persidsky Y, Heilman D, Haorah J, Zelivyanskaya M, Persidsky R, Weber GA (2006). Rho-mediated regulation of tight junctions during monocyte migration across the blood-brain barrier in HIV-1 encephalitis (HIVE). Blood.

[140] Ma T, Liu L, Wang P, Xue Y (2012). Evidence for involvement of ROCK signaling in bradykinin-induced increase in murine blood-tumor barrier permeability. J Neurooncol.

[141] Feng S, Zou L, Wang H, He R, Liu K, Zhu H. RhoA/ROCK-2 pathway inhibition and tight Junction protein upregulation by Catalpol suppresses Lipopolysaccaride-Induced disruption of blood-brain barrier permeability. Molecules. 2018;23(9). 10.3390/molecules23092371 PMC6225311 30227623

[142] Grothaus JS, Ares G, Yuan C, Wood DR, Hunter CJ (2018). Rho kinase inhibition maintains intestinal and vascular barrier function by upregulation of occludin in experimental necrotizing enterocolitis. Am J Physiol Gastrointest Liver Physiol.

[143] Chen Q, Wu Y, Yu Y, Wei J, Huang W (2021). Rho-kinase inhibitor hydroxyfasudil protects against HIV-1 Tat-induced dysfunction of tight junction and neprilysin/Abeta transfer receptor expression in mouse brain microvessels. Mol Cell Biochem.

[144] Fruman DA, Chiu H, Hopkins BD, Bagrodia S, Cantley LC, Abraham RT (2017). The PI3K pathway in Human Disease. Cell.

[145] Manning BD, Toker A (2017). AKT/PKB signaling: navigating the network. Cell.

[146] Saxton RA, Sabatini DM (2017). mTOR Signaling in Growth, Metabolism, and Disease. Cell.

[147] Wang J, Zhao W, Guo H, Fang Y, Stockman SE, Bai S (2018). AKT isoform-specific expression and activation across cancer lineages. BMC Cancer.

[148] Burgering BM, Coffer PJ (1995). Protein kinase B (c-Akt) in phosphatidylinositol-3-OH kinase signal transduction. Nature.

[149] Rahmani F, Ferns GA, Talebian S, Nourbakhsh M, Avan A, Shahidsales S (2020). Role of regulatory miRNAs of the PI3K/AKT signaling pathway in the pathogenesis of Breast cancer. Gene.

[150] Kohn AD, Kovacina KS, Roth RA (1995). Insulin stimulates the kinase activity of RAC-PK, a pleckstrin homology domain containing ser/thr kinase. EMBO J.

[151] Fresno Vara JA, Casado E, de Castro J, Cejas P, Belda-Iniesta C, Gonzalez-Baron M (2004). PI3K/Akt signalling pathway and cancer. Cancer Treat Rev.

[152] Sarbassov DD, Guertin DA, Ali SM, Sabatini DM (2005). Phosphorylation and regulation of Akt/PKB by the rictor-mTOR complex. Science.

[153] Maehama T, Dixon JE (1998). The Tumor suppressor, PTEN/MMAC1, dephosphorylates the lipid second messenger, phosphatidylinositol 3,4,5-trisphosphate. J Biol Chem.

[154] Bahena-Ocampo I, Espinosa M, Ceballos-Cancino G, Lizarraga F, Campos-Arroyo D, Schwarz A (2016). miR-10b expression in Breast cancer stem cells supports self-renewal through negative PTEN regulation and sustained AKT activation. EMBO Rep.

[155] Dillon LM, Miller TW (2014). Therapeutic targeting of cancers with loss of PTEN function. Curr Drug Targets.

[156] Andjelkovic M, Jakubowicz T, Cron P, Ming XF, Han JW, Hemmings BA (1996). Activation and phosphorylation of a pleckstrin homology domain containing protein kinase (RAC-PK/PKB) promoted by serum and protein phosphatase inhibitors. Proc Natl Acad Sci U S A.

[157] Brognard J, Sierecki E, Gao T, Newton AC (2007). PHLPP and a second isoform, PHLPP2, differentially attenuate the amplitude of akt signaling by regulating distinct akt isoforms. Mol Cell.

[158] Risso G, Blaustein M, Pozzi B, Mammi P, Srebrow A (2015). Akt/PKB: one kinase, many modifications. Biochem J.

[159] Xu F, Na L, Li Y, Chen L (2020). Roles of the PI3K/AKT/mTOR signalling pathways in neurodegenerative Diseases and tumours. Cell Biosci.

[160] Cross DA, Alessi DR, Cohen P, Andjelkovich M, Hemmings BA (1995). Inhibition of glycogen synthase kinase-3 by insulin mediated by protein kinase B. Nature.

[161] Hussain AR, Ahmed SO, Ahmed M, Khan OS, Al Abdulmohsen S, Platanias LC (2012). Cross-talk between NFkB and the PI3-kinase/AKT pathway can be targeted in primary effusion Lymphoma (PEL) cell lines for efficient apoptosis. PLoS ONE.

[162] Webb AE, Brunet A (2014). FOXO transcription factors: key regulators of cellular quality control. Trends Biochem Sci.

[163] Du K, Montminy M (1998). CREB is a regulatory target for the protein kinase Akt/PKB. J Biol Chem.

[164] Cong X, Li S, Zhang Y, Zhu Z, Wang Y, Song S (2019). The combination of preoperative fibrinogen and neutrophil-lymphocyte ratio is a predictive prognostic factor in esophagogastric junction and upper gastric cancer. J Cancer.

[165] Li N, Neu J (2009). Glutamine deprivation alters intestinal tight junctions via a PI3-K/Akt mediated pathway in Caco-2 cells. J Nutr.

[166] Lv Y, Liu W, Ruan Z, Xu Z, Fu L. Myosin IIA Regulated Tight Junction in Oxygen Glucose-Deprived Brain Endothelial Cells Via Activation of TLR4/PI3K/Akt/JNK1/2/14-3-3epsilon/NFkappaB/MMP9 Signal Transduction Pathway. Cell Mol Neurobiol. 2019;39(2):301 – 19. 10.1007/s10571-019-00654-y PMC11479604 30666520

[167] Nusrat A, Chen JA, Foley CS, Liang TW, Tom J, Cromwell M (2000). The coiled-coil domain of occludin can act to organize structural and functional elements of the epithelial tight junction. J Biol Chem.

[168] Sheth P, Basuroy S, Li C, Naren AP, Rao RK (2003). Role of phosphatidylinositol 3-kinase in oxidative stress-induced disruption of tight junctions. J Biol Chem.

[169] Barber DF, Alvarado-Kristensson M, González-García A, Pulido R, Carrera AC. PTEN Regulation, a Novel Function for the p85 Subunit of Phosphoinositide 3-Kinase. Science's STKE. 2006;2006(362):pe49. 10.1126/stke.3622006pe49 17119157

[170] Gonzalez-Garcia A, Garrido E, Hernandez C, Alvarez B, Jimenez C, Cantrell DA (2002). A new role for the p85-phosphatidylinositol 3-kinase regulatory subunit linking FRAP to p70 S6 kinase activation. J Biol Chem.

[171] Cuevas BD, Lu Y, Mao M, Zhang J, LaPushin R, Siminovitch K (2001). Tyrosine phosphorylation of p85 relieves its inhibitory activity on Phosphatidylinositol 3-Kinase. J Biol Chem.

[172] Taniguchi CM, Winnay J, Kondo T, Bronson RT, Guimaraes AR, Alemán JO (2010). The phosphoinositide 3-Kinase Regulatory Subunit p85α can exert Tumor suppressor properties through negative regulation of growth factor signaling. Cancer Res.

[173] Mao X, Hu H, Tang J, Chen D, Yu B (2016). Leucine increases mucin 2 and occludin production in LS174T cells partially via PI3K-Akt-mTOR pathway. Anim Nutr.

[174] Ko JH, Nam D, Um JY, Jung SH, Sethi G, Ahn KS. Bergamottin suppresses Metastasis of Lung Cancer cells through abrogation of Diverse Oncogenic Signaling cascades and epithelial-to-mesenchymal transition. Molecules. 2018;23(7). 10.3390/molecules23071601 PMC6100248 30004418

[175] Wu F, Chen Z, Tang C, Zhang J, Cheng L, Zuo H (2017). Acid fibroblast growth factor preserves blood-brain barrier integrity by activating the PI3K-Akt-Rac1 pathway and inhibiting RhoA following traumatic brain injury. Am J Transl Res.

[176] Wang ZG, Cheng Y, Yu XC, Ye LB, Xia QH, Johnson NR (2016). bFGF protects against blood-brain barrier damage through Junction protein regulation via PI3K-Akt-Rac1 pathway following traumatic Brain Injury. Mol Neurobiol.

[177] Eguchi H, Akizuki R, Maruhashi R, Tsukimoto M, Furuta T, Matsunaga T (2018). Increase in resistance to anticancer Drugs involves occludin in spheroid culture model of lung adenocarcinoma A549 cells. Sci Rep.

[178] He S, Guo Y, Zhao J, Xu X, Song J, Wang N (2019). Ferulic acid protects against heat stress-induced intestinal epithelial barrier dysfunction in IEC-6 cells via the PI3K/Akt-mediated Nrf2/HO-1 signaling pathway. Int J Hyperthermia.

[179] Zhuang Y, Wu H, Wang X, He J, He S, Yin Y (2019). Resveratrol attenuates oxidative stress-Induced Intestinal Barrier Injury through PI3K/Akt-Mediated Nrf2 signaling pathway. Oxid Med Cell Longev.

[180] Diaz-Canestro C, Merlini M, Bonetti NR, Liberale L, Wust P, Briand-Schumacher S et al. Corrigendum to ‘Sirtuin 5 as a novel target to blunt blood-brain barrier damage induced by cerebral ischemia/reperfusion injury’ [Int. J. Cardiol. 260 (2018) 148–155]. Int J Cardiol. 2018;271:405. 10.1016/j.ijcard.2017.12.060 29622432

[181] Contreras X, Barboric M, Lenasi T, Peterlin BM (2007). HMBA releases P-TEFb from HEXIM1 and 7SK snRNA via PI3K/Akt and activates HIV transcription. PLoS Pathog.

[182] Chugh P, Bradel-Tretheway B, Monteiro-Filho CM, Planelles V, Maggirwar SB, Dewhurst S (2008). Akt inhibitors as an HIV-1 infected macrophage-specific anti-viral therapy. Retrovirology.

[183] Zhou F, Liu X, Gao L, Zhou X, Cao Q, Niu L (2019). HIV-1 Tat enhances purinergic P2Y4 receptor signaling to mediate inflammatory cytokine production and neuronal damage via PI3K/Akt and ERK MAPK pathways. J Neuroinflammation.

[184] Yang B, Singh S, Bressani R, Kanmogne GD (2010). Cross-talk between STAT1 and PI3K/AKT signaling in HIV-1-induced blood-brain barrier dysfunction: role of CCR5 and implications for viral neuropathogenesis. J Neurosci Res.

[185] Antonetti DA, Barber AJ, Hollinger LA, Wolpert EB, Gardner TW (1999). Vascular endothelial growth factor induces rapid phosphorylation of tight junction proteins occludin and zonula occluden 1. A potential mechanism for vascular permeability in diabetic retinopathy and tumors. J Biol Chem.

[186] Li R, Qi Y, Jiang M, Zhang T, Wang H, Wang L (2019). Primary tumor-secreted VEGF induces vascular hyperpermeability in premetastatic lung via the occludin phosphorylation/ubiquitination pathway. Mol Carcinog.

[187] Muthusamy A, Lin CM, Shanmugam S, Lindner HM, Abcouwer SF, Antonetti DA (2014). Ischemia-reperfusion injury induces occludin phosphorylation/ubiquitination and retinal vascular permeability in a VEGFR-2-dependent manner. J Cereb Blood Flow Metab.

[188] Liu X, Dreffs A, Diaz-Coranguez M, Runkle EA, Gardner TW, Chiodo VA (2016). Occludin S490 Phosphorylation regulates vascular endothelial growth factor-Induced Retinal neovascularization. Am J Pathol.

[189] Runkle EA, Sundstrom JM, Runkle KB, Liu X, Antonetti DA (2011). Occludin localizes to Centrosomes and modifies mitotic entry. J Biol Chem.

[190] Wang LF, Li X, Gao YB, Wang SM, Zhao L, Dong J (2015). Activation of VEGF/Flk-1-ERK Pathway Induced Blood-Brain Barrier Injury after microwave exposure. Mol Neurobiol.

[191] Serrano VB, Montoya JL, Campbell LM, Sundermann EE, Iudicello J, Letendre S (2021). The relationship between vascular endothelial growth factor (VEGF) and amnestic mild cognitive impairment among older adults living with HIV. J Neurovirol.

[192] Sporer B, Koedel U, Paul R, Eberle J, Arendt G, Pfister HW (2004). Vascular endothelial growth factor (VEGF) is increased in serum, but not in cerebrospinal fluid in HIV associated CNS Diseases. J Neurol Neurosurg Psychiatry.

[193] Carmeliet P, Storkebaum E (2002). Vascular and neuronal effects of VEGF in the nervous system: implications for neurological disorders. Semin Cell Dev Biol.

[194] Ascherl G, Hohenadl C, Schatz O, Shumay E, Bogner J, Eckhart L (1999). Infection with human immunodeficiency virus-1 increases expression of vascular endothelial cell growth factor in T cells: implications for acquired immunodeficiency syndrome-associated vasculopathy. Blood.

[195] Sharma AL, Wang H, Zhang Z, Millien G, Tyagi M, Hongpaisan J (2022). HIV promotes neurocognitive impairment by damaging the hippocampal microvessels. Mol Neurobiol.

[196] McKenzie JAG, Riento K, Ridley AJ (2006). Casein kinase Iε associates with and phosphorylates the tight junction protein occludin. FEBS Lett.

[197] Smales C, Ellis M, Baumber R, Hussain N, Desmond H, Staddon JM (2003). Occludin phosphorylation: identification of an occludin kinase in brain and cell extracts as CK2. FEBS Lett.

[198] Schubert U, Henklein P, Boldyreff B, Wingender E, Strebel K, Porstmann T (1994). The human immunodeficiency virus type 1 encoded vpu protein is phosphorylated by casein kinase-2 (CK-2) at positions Ser52 and Ser56 within a predicted alpha-helix-turn-alpha-helix-motif. J Mol Biol.

[199] Ohtsuki K, Maekawa T, Harada S, Karino A, Morikawa Y, Ito M (1998). Biochemical characterization of HIV-1 rev as a potent activator of casein kinase II in vitro. FEBS Lett.

[200] Hirase T, Staddon JM, Saitou M, Ando-Akatsuka Y, Itoh M, Furuse M (1997). Occludin as a possible determinant of tight junction permeability in endothelial cells. J Cell Sci.

[201] Kim KA, Kim D, Kim JH, Shin YJ, Kim ES, Akram M (2020). Autophagy-mediated occludin degradation contributes to blood-brain barrier disruption during ischemia in bEnd.3 brain endothelial cells and rat ischemic Stroke models. Fluids Barriers CNS.

[202] Sugiyama S, Sasaki T, Tanaka H, Yan H, Ikegami T, Kanki H (2023). The tight junction protein occludin modulates blood-brain barrier integrity and neurological function after ischemic Stroke in mice. Sci Rep.

[203] Rempe RG, Hartz AMS, Soldner ELB, Sokola BS, Alluri SR, Abner EL (2018). Matrix metalloproteinase-mediated blood-brain barrier dysfunction in Epilepsy. J Neuroscience: Official J Soc Neurosci.

[204] Zengil S, Laloglu E (2023). Evaluation of serum Zonulin and occludin levels in bipolar disorder. Psychiatry Invest.

[205] Bilgic A, Ferahkaya H, Karagoz H, Kilinc I, Energin VM (2023). Serum claudin-5, claudin-11, occludin, vinculin, paxillin, and beta-catenin levels in preschool children with autism spectrum disorder. Nord J Psychiatry.

